# Distance Education among Italian Teachers: Differences and Experiences

**DOI:** 10.1007/s10639-022-11008-5

**Published:** 2022-03-30

**Authors:** Laura Menabò, Grace Skrzypiec, Alessandra Sansavini, Antonella Brighi, Annalisa Guarini

**Affiliations:** 1 Department of Psychology “Renzo Canestrari”, University of Bologna, Viale Berti Pichat 5, 40127 Bologna, Italy; 2 College of Education Psychology and Social Work, Flinders University, Sturt Rd, Bedford Park, Adelaide, South Australia 5001 Australia; 3 Faculty of Education, Free University of Bozen-Bolzano, Via Ratisbona 16, 39042 Brixen-Bressanone, Italy

**Keywords:** Distance education, Pre-service teachers, Primary school teachers, Secondary school teachers, Mixed-method, COVID-19

## Abstract

The successful integration of technology in teaching is a key component of education. Although prior research highlighted factors fostering the use of technology by teachers, few studies focused on whether these factors vary among teachers of different grade levels and subjects. Moreover, no studies have investigated personal experiences related to distance education among a large sample of teachers. To address these gaps, the present mixed-method study sought to examine whether factors promoting distance education varied among Italian teachers of different grade levels and subjects. A further aim was to explore experiences of teachers using distance education. The sample involved 357 Italian teachers and preservice teachers who completed an online questionnaire during the COVID-19 pandemic that also contained open-ended questions. Findings indicated that teaching self-efficacy was greater in pre-service and primary teachers, while facilitating conditions were greater in humanities and science secondary teachers. The perceived ease of use of technology and technology for pedagogy skills were more pronounced among science secondary teachers. Advanced technology skills were lower in humanities secondary teachers while the behavioural intention to use technology was greatest among pre-service teachers. Four themes emerged from the qualitative study of teachers’ insights. These included positive and negative aspects of using technology, the relationship with students, the versatility of distance education, and the quality of lessons. This study underscores the need to address training based on different teachers’ grade levels and subjects, and to focus on the emerging themes to better integrate the use of technology in schools.

The importance of successfully integrating technology in teaching is not a new topic for policymakers and educational researchers (Voogt et al., 2013). The OECD (2015) and European Commission (2016) for example, stated that member states should foster the development of new digitalized learning environments to ensure national education systems stay up to date (Salmieri, 2019). In addition, several studies reported that the integration of technology in instruction is an essential ingredient for student success in the 21st-century (Foster et al., 2011; Harter, 2011; Washbon, 2012). However, the past two years have revealed the difficulty of integrating technology in the education system worldwide. Indeed, the spread of COVID-19 forced several countries, like Italy, to shift educational activities to digital environments, fueling uncertainties and disagreement about how to implement it (Pellegrini & Maltinti, 2020). Thus, online learning has became the main challenge not only for universities, where distance education was already familiar, but also for primary and secondary schools. In this context, it becomes crucial to understand how factors can promote technology to support distance education such as that needed during the COVID-19 pandemic and to describe the associated experiences, feelings, and perceived challenges. Moreover, even if previous literature has highlighted differences in integrating technology based on different school policies, literature on this topic during COVID-19 seems scarce, requiring further investigation.

## Factors Promoting Distance Education

Despite several variables influencing the decision to adopt technology in schools, some of them, such as teacher self-efficacy, facilitating conditions, perceived ease of use of technology, teacher digital skills and behavioural intention to use technology, seem to be particularly relevant (Scherer et al., 2019; Teo, 2009).

Teaching self-efficacy (i.e., the belief and confidence to implement good teaching in the classroom; Christophersen et al., 2016) and online teaching self-efficacy (i.e., self-efficacy related to technology; Anderson et al., 2011; Banas & York, 2014) are both crucial in education (Brouwers & Tomic, 2003; Henson, 2011; Valtonen et al., 2015). Since the pandemic, many studies on teaching self-efficacy and online teaching self-efficacy have been conducted among different countries, revealing a general decrease in teachers’ self-efficacy (Cataudella et al., 2021; Pellerone, 2021; Pressley & Ha, 2021; Takunyaci, 2021) and a general increase in online teaching self-efficacy. Ma et al. (2021), for example, found an increase in online teaching self-efficacy in Chinese teachers during the COVID-19 pandemic, while Košir et al. (2020) showed that Serbian teachers with high online teaching self-efficacy had positive attitudes towards distance education and experienced less stress in using technologies.

Facilitating conditions, (i.e. the factors in the environment that influence a person’s perception of how easy or difficult it is to perform a task; Teo, 2009), are critical indicators for promoting new technology both in preservice and inservice teachers (Teo, 2009, 2011). Sangeeta and Tandon (2020) analyzed facilitating conditions in a sample of 643 Indian school teachers during the COVID-19 pandemic, and found a significant positive impact on behavioural intentions to use technology.

Perceived ease of use of technology, defined as the degree to which the potential user expects the system to be effortless (Davis et al., 1989), determines a teachers’ use of technology (Hu et al., 2003). During the COVID-19 pandemic, Rahayu and Wirza (2020) analyzed the perceived ease of use of technology in 102 Indonesian teachers, and revealed a positive perception of the usefulness and ease of use of distance education. Alhumaid et al. (2020) analyzed Pakistani university instructors’ perceptions and showed that perceived usefulness affected the positive relationship between technology acceptance and distance education.

Furthermore, teachers’ acquisition of new skills and practices related to technology use is another crucial aspect influencing the decision to adopt technology (Lee et al., 2019). Teachers need to be equipped with different digital skills since they need to use digital technologies with well-founded pedagogy to enhance students’ learning and to facilitate their digital competence (Krumsvik, 2014; Redecker, 2017). Different types of skills, including basic and advanced skills, and the technology for pedagogy, are required (Hatlevik, 2017; Teo & Milutinovic, 2015; Tondeur et al., 2018). However, teachers do not seem to be suitably trained to acquire digital competencies in digital environments. Indeed, studies during COVID-19 highlighted the urgent need to improve technology skills and competencies among teachers (Portillo et al., 2020; Trubavina et al., 2021).

Another factor considered relevant in the extant literature is the behavioural intention to use technology, where it is defined as “a cognitive process of individuals’ readiness to perform specific behaviour …[which] … is an immediate antecedent of usage behaviour” (Abbasi et al., 2011, p 36). Behavioural intention is the key factor determining the success of a system (Abdullah & Ward, 2016; Armenteros et al., 2013; Chang et al., 2017) and it is considered the most important predictor of the actual use of technology (Teo, 2011). During the COVID-19 pandemic, a great deal of research analyzed this construct as the primary outcome, showing interesting results with regard to how the factors described above interacted with each other to influence behavioural intention (Mailizar et al., 2021; Menabò et al., 2021; Sangeeta & Tandon, 2020). Although these studies have merit and have showed new ways in which the factors above interacted, none of them considered all factors together.

## Challenges and Experiences in Distance Education

Challenges and experiences related to the use of distance education had been widely explored before the COVID-19 pandemic but mainly in tertiary education (for a literature review, see Carrillo & Flores, 2020; Kebritchi et al., 2017) while literature was scarce concerning secondary schools. However, due to the increased use of distance education during the COVID-19 pandemic, some studies have examined distance education in primary and secondary schools using a qualitative approach.

Atmojo and Nugroho (2020) interviewed 16 Indonesian upper secondary teachers to reveal the critical financial condition of many students’ families that impeded distance education. They found that many students lacked smartphones, Internet quota, and stable Internet connections. Similar issues were reported by three upper secondary school teachers in Zambia (Sintema, 2020), where they expressed concern for their students’ academic performance because of the lack of technological devices. Difficulties with Internet access and lack of infrastructure, classroom management and human resources also emerged as concerns among 65 Turkish teachers in a study by Sari and Nayir (2020).

A significant challenge for teachers during the COVID-19 pandemic concerned their skills in using technology for education. Teachers reported not being ready for the distance education process, claiming the need for support and distance education training (Sari & Nayir, 2020). In particular, 50 Turkish teachers reported negative views of their online competency due to their non-creative traits and inability to use interactive resources (Koçoglu & Tekdal, 2020). Similar considerations emerged among six North American primary school educators, who described struggling to learn to use technology and for providing meaningful but socially distant learning experiences (Anderson & Hira, 2020). In a similar vein, Indonesian upper secondary teachers described a general lack of preparation and planning for distance education (Atmojo & Nugroho, 2020). A further consideration reported by teachers concerned the difficulty of evaluating and monitoring students and designing online courses based on available resources (Hebebci et al., 2020; Niemi & Kousa, 2020; Sari & Nayır, 2020).

Beyond the concerns associated with distance education, teachers also reported benefits and positive aspects, such as the possibility of interacting with students even if in a period of emergency, and the possibility of creating meaningful and entertaining lessons thanks to the use of technology and staying at home (Danchikov et al., 2021; Hebebci et al., 2020; Niemi & Kousa, 2020).

While the qualitative studies described above provided an in-depth analysis of teachers’ challenges and experiences in distance education during the COVID-19 pandemic, they involved a low number of participants, and posed limitations in the generalization of results. No studies have investigated positive and negative experiences associated with distance education among a large Italian sample of teachers, using interviews or questionnaires with open-ended questions.

## Differences Among Teacher School Grade Level and Subject Area

Differences in the integration of technology among teachers of different grade levels’ and subjects have been studied before the spread of COVID-19, showing interesting results. Kindergarten teachers, for example, were found to be less prone to use technology than higher grade teachers, due to kindergarten children’s limited reading and writing abilities (Antonietti & Giorgetti, 2006; Cordes & Miller, 2000; Magen-Nagar & Firstater, 2019). By contrast, preservice teachers, who are often part of the Net-generation and actively use technology in everyday living (Tapscott, 2008), have shown very positive attitudes towards the use of technology (Koc & Gulyagci, 2013; McGarr & Gavaldon, 2018; Şad & Göktaş, 2014). Al-Awidi and Alghazo (2012) examined the role of pre-service teachers teaching experiences in their self-efficacy of technology integration and found that teaching experiences, especially mastery and vicarious experiences, significantly affected their self-efficacy in technology integration. That is, the teaching experiences of pre-service teachers helped them foster their self-efficacy on technology integration since they were able to put into practice what they had learned during their teacher education.

Differences between teachers are considered to be related to different pedagogical beliefs in using technology, defined as the teacher’s own understanding about teaching and learning (Ertmer et al., 2010). Pedagogical beliefs have been found to be strong predictors of the educational use of technology (Ertmer et al., 2015; Tondeur et al., 2021). Specifically, teachers seem to select technological applications that align with their existing beliefs about “good education” (Tondeur et al., 2021).

Research has shown that technology use can also differ among teachers of different subjects, especially science and humanities teachers, since their different pedagogical beliefs relate to technology use (Karaseva et al., 2015). One reason may be the different perspectives in which teachers are introduced to technology in their training and academic paths (Karaseva et al., 2015) since much emphasis is on the importance of technology for science teachers (Hammond et al., 2011). Another aspect is the perceived subject nature and how the new technology fits existing subject practices and content. For example, John and La Velle (2004) argued that science and mathematics teachers held relatively open attitudes towards the potential of technology to transform teaching, which is consistent with the role of mathematics in the evolution of digital technologies. Literature teachers, on the contrary, were found to be more anxious about “losing the core features and values” of their subject, classroom discussion, and use of printed text (Hennessy et al., 2005). However, other studies have shown contrasting results, revealing that mathematics teachers were less likely to integrate technology in classrooms than literature teachers because mathematics requires repetitive practices to master knowledge, and technology was not considered useful and important for learning mathematics (Howard & Maton, 2011).

Although these differences could affect how teachers approach themselves to distance education, an investigation conducted on “Scopus” and “Web of Science” databases searches showed that only a few published works have evaluated the differences among grade levels and subjects during the COVID-19 pandemic, and these have shown contrasting results. Giovannella and colleagues (2020) investigated a large sample of Italian teachers and found that upper secondary school teachers had higher readiness to switch to online education compared to their colleagues at other school levels. Another Italian survey (Scarpellini et al., 2021) showed that parents perceived lessons as less organized and routines more unstable for primary school students, while secondary school teachers were more prepared to implement distance learning. By contrast, Alea and colleagues (2020), who examined Philippine teachers from different grade levels, did not find any differences among the subjects taught and the teachers’ level of education.

## The Present Study

The present research investigated distance education in 2 months during the COVID-19 pandemic, as described by Italian inservice and preservice teachers. Indeed, although the studies presented so far have provided meaningful insights about the implementation of distance education in the emergency period of COVID-19, we believe that some questions still need answers. Specifically, the first research question was: “How the factors promoting technology vary in function of the teacher's grade levels and subject areas?”. As described above, only three studies have been carried out on the differences among school grade levels and subject areas in the readiness to use technology, revealing contrasting results (Alea et al., 2020; Giovannella et al., 2020; Scarpellini et al., 2021). We used a quantitative approach to respond to the first research question. In detail, we compared a large sample of preservice and inservice teachers of different school grade levels and subjects, analysing differences on several variables promoting distance education, such as teacher self-efficacy, online teaching self-efficacy, facilitating conditions, perceived ease of use, basic technology skills, advanced technology skills, technology for pedagogy and behavioral intention to use technology.

The second research question was “What are the personal experiences, both positive and negative, feelings and concerns about distance education during the COVID-19 pandemic among teachers of different school levels and subject areas?” Previous studies focusing on primary or secondary school teachers involved only small groups and did not consider differences among teachers of different school levels and subjects (Atmojo & Nugroho, 2020; Hebebci et al., 2020; Niemi & Kousa, 2020; Sari & Nayır, 2020) We used a qualitative approach to investigate the second research question among a large group of preservice and inservice teachers of different school grade levels and subjects.

Thus, the present study adopted a mixed-method design, which increased validity in the findings and gained a deeper understanding, as suggested by previous research (Hurmerinta-Peltomäki & Nummela, 2006; Östlund et al., 2011). Although some studies including mixed-method research designs have been published, many of them have focused on tertiary education (Crowe et al., 2021; Popa et al., 2020), while few studies have used a mixed-method approach in primary and secondary schools (Cardullo et al., 2021; Hussein et al., 2021). To our knowledge, no studies have used a mixed-method research method to investigate Italian teachers’ experiences of distance education during the COVID-19 lockdown. Such a study is particularly pertinent among Italian teachers, as they endured a long period of distance education during the COVID-19 lockdown, which lasted from March 2020 till May 2021, with some periods of interruption. In addition, although preservice teachers were not directly involved in distance education, we felt that the comparison among inservice and preservice teachers might be interesting in understanding how the future generation of teachers view technology and their opinions and feelings about distance education in line with other studies (Chen et al., 2019; Hughes et al., 2020; Ismailos et al., 2022;Tajeddin and Alemi, 2019; Ursavaş et al., 2019).

## Method

### Participants

In the present study, 357 inservice and preservice teachers completed an online questionnaire available on QUALTRICS between 15 May and 10 July 2020, during the school closure period for the COVID-19 lockdown in Italy. The respondents were recruited online through convenience sampling (e.g., researcher contacts, survey advertising on social networks, etc.).

The sample comprised 27% (*n*= 95) preservice teachers, 22% (*n*= 80) primary school teachers, 28% (*n*= 99) upper secondary school teachers of humanities (literature, history, geography), and 23% (*n*= 83) upper secondary school teachers of STEM subjects (maths, science, technology). The majority of participants were female and located in Northern Italy and ranged in ages from 21 to 61+ (see Sociodemographic information in Table 1).

**Table 1:** Sociodemographic Characteristics of Participants

Characteristics	Preservice teachers		Primary teachers		Humanities teachers		Science teachers		Total sample	
	n	%	n	%	n	%	n	%	n	%
Gender										
Female	76	94	70	96	70	81.5	40	59.5	256	83.5
Male	5	6	3	4	16	18.5	27	40.5	51	16.5
Age										
21-30	68	84	15	20.5	3	3.5	5	7.5	91	29.5
31-40	8	10	8	11	6	7	5	7.5	27	9
41-50	5	6	20	27.5	21	24.5	22	33	68	22
51-60	0	0	26	35.5	39	45	23	34	88	29
+61	0	0	4	5.5	17	20	12	18	33	10.5
Location										
Northern-Italy	69	84	64	87	55	65	46	70	234	77
Central-Italy	13	16	7	10	23	27	16	25	59	19
South-Italy	0	0	2	3	7	8	3	5	12	4

### Measures

An online battery of questionnaires, including standardized scales, open-ended questions, and demographic information was administered. The entire questionnaire took about 10-15 minutes to complete.

#### Teaching Self-efficacy

The subjective sense of success in teaching was assessed using the Teachers’ Sense of Efficacy Scale (TSES; Tschannen-Moran & Hoy, 2001). In this scale, teaching is conceptualized as a complex activity and represents teachers’ efficacy as a multi-faceted construct: efficacy in classroom management (CM), efficacy in promoting student engagement (SE), and efficacy in using instructional strategies (IS).

We decided to include the short form of SE and IS scales, for a total of 8 questions (e.g “How much can you assist families in helping their children do well in school?”, “To what extent can you provide an alternative explanation or example when students are confused?”). Each item was scored on a 9-point Likert-type scale from 1 (not at all) to 9 (a great deal). These scales presented an excellent internal consistency with a Cronbach’s alpha of 0.88 and 0.93, respectively.

#### Online Teaching Self-efficacy

Online teaching self-efficacy was evaluated using an adaptation of the Teacher Sense of Efficacy Scale (TSES, Tschannen-Moran & Hoy, 2001). The questionnaire had already been modified by Robinia and Anderson (2010) to investigate online teaching efficacy. However, their questionnaire targeted nurse educators in academic institutions. Thus, we decided to newly adapt the two scales (SE and IS) to assess teaching self-efficacy contextualized for an online environment in primary and secondary schools. The final questionnaire comprised 8 items (e.g. “How much can you assist families online in helping their children do well in school?”, “To what extent can you provide online an alternative explanation or example when students are confused?”). Each item was scored on a 9-point Likert-type scale from 1 (not at all) to 9 (a great deal). Cronbach’s alpha was respectively 0.91 and 0.89.

#### Facilitating Conditions

Facilitating conditions were assessed using the “facilitating conditions scale” (Teo, 2011), which comprised three questions (“When I encounter difficulties in using technology, a specific person is available to assist”; “When I encounter difficulties in using technology, I know where to seek assistance”; “When I encounter difficulties in using technology, I am given timely assistance”) The scale was assessed on a 7-point Likert scale (1 = strongly disagree and 7 = strongly agree). Cronbach’s alpha was 0.85.

#### Perceived Ease of Use of Technology

Perceived ease of use of technology was assessed using a 5-item scale derived from Teo’s (2011) study (e.g “Learning to use technology is easy for me”, “My interaction with technology does not require much effort”). The scale was assessed on a 7-point Likert scale (1 = strongly disagree and 7 = strongly agree). Cronbach’s alpha was 0.93.

#### Basic Technology Skills

Basic technology skills were evaluated using a 3-item scale (e.g., “I am able to use the internet to search for information and resources”; “I am able to use Presentation Software (e.g. Microsoft Powerpoint) for classroom delivery”; Teo, 2009). The scale was assessed on a 7-point Likert scale (1= strongly disagree and 7= strongly agree). Cronbach’s alpha was 0.70.

#### Advanced Technology Skills

Advanced technology skills were evaluated using a 3-item scale proposed by Teo (2009; “I am able to use website Editors, e.g. Microsoft FrontPage, Macromedia Dreamweaver, to create and/or modify web pages.”, I am able to use video editing software, e.g. Microsoft MovieMaker, Adobe Premier, UleadVideoStudio”). The scale was assessed on a 7-point Likert scale (1= very strongly disagree and 7= very strongly agree). Cronbach’s alpha was 0.84

#### Technology for Pedagogy

The ability to use technology for pedagogical purposes was evaluated by a 4-item scale (“I search, evaluate and select appropriate technological resources to support lesson activities; “I am able to adopt and adapt given IT-based learning activities”; “I can manage technology-based learning activities in a computer laboratory”; “I am able to adopt and adapt activities that incorporate the use of technology to assess pupils’ learning and provide immediate and constructive feedback”; Teo, 2009). The scale was assessed on a 7-point Likert scale (1= very strongly disagree and 7= very strongly agree). Cronbach’s alpha was 0.87.

#### Behavioral Intention to Use Technology

The behavioral intention to use technology was assessed using a 3-item scale (e.g., “I intend to continue to use technology in the future”; “I expect I would use technology in the future”; Teo, 2011). The scale was assessed on a 7-point Likert scale (1 = very strongly disagree and 7 = very strongly agree). Cronbach’s alpha was 0.94.

#### Positive and Negative Aspects of Distance Teaching

To gain a deeper understanding of the thoughts and opinions regarding the use of distance teaching, two open-ended questions were included in the questionnaire. The first question (“What do you think are the positive aspects in distance teaching?”) aimed at investigating the positive aspects in the use of distance teaching. The second question (“What do you think are the negative aspects in distance teaching?”) aimed at shedding light on the difficulties encountered by teachers in the use of distance teaching.

#### Sociodemographic Information

Finally, sociodemographic information including age, gender, location (region), school grade, and subject taught, was collected.

### Ethics

Formal approval for the study was provided by the Bioethics Committee. In the informed consent, participants were provided with information about the purpose of the research and the procedures; the benefits/risks of participating in this study; the rights to decline to participate and to withdraw from the research without consequences according to the Declaration of Helsinki. Participants did not receive incentives or benefits for their participation.

### Data Analysis

Quantitative data were analyzed through one-way ANOVAs in SPSS 26 to understand differences between groups (preservice teachers, primary teachers, secondary school teachers of humanities, and secondary school teachers of science subjects) and when a significant difference was found post-hoc tests were performed (Bonferroni). Qualitative data were analyzed through text analysis and content analysis on Nvivo 11, following the phases suggested by Elo and Kyngäs (2008). Content analysis is a method that is effective in classification, edition, and comparison of texts to make theoretical inferences. The answers were evaluated in detail by two independent researchers (first and last authors of the article) to check inter-rater reliability. Then, the researchers created codes reflecting the opinions of the participants. Subsequently, related codes were grouped, and themes were created. The process was concluded by interpreting the themes and codes associated with each other.

## Results

### Quantitative Findings

Concerning teaching self-efficacy, one-way ANOVA yielded a significant group effect, *F* (3,317) = 5.49, *p*= .001 (Table 2). Bonferroni post-hoc test revealed that preservice teachers (*M*= 54.34) showed a greater level of self-efficacy than humanities teachers (*M*= 50.17, *se*= 1.37, *p*= .02) while primary teachers (*M*= 54.50) had a higher level of self-efficacy compared to both humanities and science teachers (*M*= 50.17, *se*= 1.40, *p*= .015; *M*= 50.48, *se*=1.49, *p*=.046 respectively).

**Table 2:** Means, Standard Deviations, and One-way Analysis of Variance of the Variables in Different Groups

Measure	*n*	Preservice teachers (A)	Primary teachers (B)	Humanities teachers (C)	Science teachers (D)	F	η^2^	Post-hoc (Bonferroni)
		*M (SD)*	*M (SD)*	*M (SD)*	*M (SD)*			
Self-efficacy	321	54.34 (8.00)	54.50 (9.47)	50.17 (9.97)	50.48 (8.69)	5.49**	.05	A,B>C B>D
Online self-efficacy	325	43.40 (10.21)	43.14 (12.94)	42.11 (11.84)	44.33 (11.45)	0.49	.005	-
Facilitating conditions	320	13.85 (4.30)	12.50 (4.75)	14.87 (4.60)	15.39 (4.37)	6.08***	.06	C,D>B
Perceived ease of use	316	25.16 (6.67)	22.57 (7.83)	23.32 (7.38)	26.40 (6.35)	4.40**	.04	D>B
Basic technology skills	321	19.38 (2.38)	18.45 (3.28)	18.31 (3.06)	19.52 (2.33)	3.88*	.04	-
Advanced technology skills	307	10.48 (4.82)	11.67 (5.43)	8.11 (4.85)	11.00 (5.60)	7.09***	.07	A,B,D>C
Technology for pedagogy	303	19.44 (4.37)	19.43 (6.06)	17.30 (6.40)	20.68 (4.99)	4.95**	.05	D>C
Behavioural intention to use	320	17.95 (2.67)	15.60 (5.15)	15.82 (4.32)	15.78 (4.51)	5.73**	.05	A>B,C,D

*p < .05, ** p < .01, ***p < .001

By contrast, no differences among groups were found in online teaching self-efficacy [*F* (3,321)= 0.49, *p*= .68].

With respect to the facilitating conditions, a significant group effect was found, *F* (3, 316) = 6.08, *p*<.001 (Table 2). Bonferroni post-hoc tests revealed that humanities teachers (*M*= 14.87) and science teachers (*M*= 15.39) showed higher facilitating conditions than primary teachers (*M*= 12.50, *se*= 0.70, *p*= .005; *se*= 0.74, *p*= .001, respectively).

A significant group effect emerged on the perceived ease of use of technology, *F* (3, 312) = 4.40, *p=* .005 (Table 2), with lower scores among primary teachers (*M*= 22.57) compared to science teachers (*M*= 26.40; *se*= 1.19, *p*= .009).

The ANOVA analysis on basic technology skills revealed a significant group effect, *F* (3, 317) = 3.88, *p*= .01, Table 2, even if differences among groups did not reach a significant level using Bonferroni post-hoc comparisons. The advanced technology skills showed a significant group effect *F* (3, 303) = 7.09, *p* < .001 (Table 2), with humanities teachers (*M*= 8.11) having lower scores than preservice teachers (*M*= 10.48, *se*= 0.73, *p*= .016), primary school teachers (*M*= 11.67, *se*= 0.83, *p* < .001) and science colleagues (*M*= 11.00, *se*= 0.85, *p*= .005).

A significant group effect was found with regard to the use of technology for pedagogy, *F* (3, 299) = 4.95, *p*= .002 (Table 2). Post-hoc test revealed lower scores among teachers of humanities (*M*= 17.30) compared to science teachers (*M*= 20.68, *se*= 0.90, *p*= .001).

Finally, ANOVA indicated a significant group effect in the behavioral intention to use technology, *F* (3, 316) = 5.73, *p*= .001 (Table 2), with the intention of preservice teachers (*M*= 17.95) significantly greater when compared to primary teachers (*M*= 15.60, *se*= 0.66, *p*= .003), teachers of humanities (*M*= 15.82, *se*= 0.64, *p*= .006) and science teachers (*M*= 15.78, *se*= 0.68, *p*= .010).

#### Qualitative Findings

**Fig. 1:**
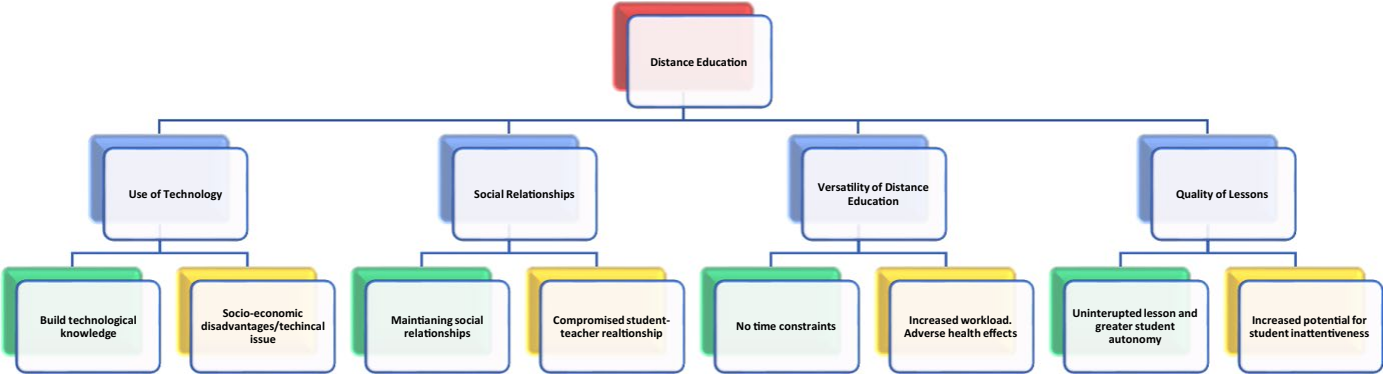
Thematic Findings of the Qualitative Approach. *Note.* The figure shows the four main themes related to distance education that emerged from the content analysis. Each theme is defined in its positive and negative meaning

Four main thematic areas emerged from the content analysis: “use of technology”; “social relationship”; “versatility of distance education”; and “quality of lessons”. The occurrence of each theme was analyzed for its positive and negative meanings. Consequently, each theme will be described in both these components, as shown in Fig 1.

#### The use of technology

Regarding the use of technology as a positive aspect (preservice teachers: 20 references, 29%; primary school teachers: 25 references, 37%; secondary school teachers of humanities and science: 12 references, 18%; 11 references, 16% respectively), distance teaching provided an opportunity for students and teachers to increase their often limited technological knowledge, as highlighted by one preservice teacher: *“Distance education permits the improvement of everyone’s computer skills, and allows students to understand the potential of technology and its use beyond its forms of entertainment such as social networks”.*

However, many negative aspects in the use of technology emerged too (preservice teachers: 20 references, 50%; primary teachers: 11 references 28%; humanities teachers: 4 references, 11%; science teachers: 4 references, 11%), implying two principal features. The first one was about social differences: some families may not have their own digital devices, and this would prevent their children from learning in the distance mode and, in turn, it could accentuate existing social differences. For example, one primary teacher wrote: *“Not every student has got powerful tools at their disposal to do online education. It would create inconvenience for connection outages, slowness, etc... and it would become difficult to resume every time a student encounters problems”.* The second negative aspect involved technical issues related to technology such as connection problems and the lack of technical skills. As one primary teacher explained *“Live video-conference lessons are not effective: connection problems, time dilatation…. And sometimes students do not have adequate devices, both for economic difficulties and lack of awareness (socio-cultural problem)”.*

#### Social relationships

Social relationships are part of school daily life. Indeed, students have the chance to learn not only academic content but also how to interact with peers and adults, and so develop their relational skills. In an emergency period, distance learning represented the only way to continue the educational path and to keep the relationship with students alive (primary teachers: 25 references, 37%; preservice teachers: 20 references, 29%; science teachers: 12 references, 18%; humanities teachers: 11 references, 16%). Indeed, *as highlighted by one primary teacher:* “*This type of school allows children to maintain a certain stability with meeting teachers and their peers, thus giving a sense of belonging and bonding”. Furthermore, another* primary teacher explained: *“Distance learning has certainly made it possible to keep the relationship between teachers and students alive, to continue their education, and to calm pupils' anxieties”*

Despite its benefit, the interaction mediated by technological devices was interpreted as a major negative factor for most of the sample, regardless of school level (preservice teachers: 42 references, 25%; primary teachers: 37 references, 23%; humanities teachers: 48 references, 30%; science teachers: 35 references, 22%). Indeed, as reported by an upper science teacher: “*The main channel of the teacher-learner relationship is the empathic and affective relationship established between the two. Distance learning inhibits, or at least limits, this relationship”. A similar expression illustrates the sentiment of most of the primary teachers in the study: “The teacher-student relationship is very compromised and less direct, the screen of a computer does not help social relationships, especially in this historical period”.*

#### Versatility of distance education

Distance education was thought to bring some advantages such as the fact that it can be used regardless of the time of the day, allowing students, who for different reasons could not attend class, to keep up with their classmates (preservice teachers: 23 references, 46%; humanities teachers: 19 references, 38%; primary teachers school: 5 references, 6%; science teachers: 3 references, 10%). As one preservice teacher explained: *“Thanks to distance learning, children who, for whatever reason, might not be able to attend school are allowed to participate in lessons”. Aligned with this sentiment one science teacher stated that "For upper secondary school students, it might be an advantage to be able to attend some classes online when they can't be physically present at school (illness, convalescence, problems due to logistics and travel)".*

The downside of this versatility, however, was that it involved a greater amount of work for teachers, and they were concerned for their own and their students’ health (humanities teachers: 13 references, 50%; preservice teachers: 8 references, 27%; primary teachers: 6 references, 19%; science teachers: 1 reference, 4%). Indeed, some teachers complained of an excessive workload involving many extra hours compared to normal classroom teaching. One humanities teacher reported that: *“Lack of awareness (even among those who belong to the school field but do not work in the classroom), respect and consideration for the enormous work that teachers must do to try to work seriously and effectively with distance teaching, much more time and effort are needed with the continuing awareness that "an indispensable piece is missing”.*

Furthermore, some teachers were concerned that prolonged use of electronic devices could lead to eye or posture problems, as one primary teacher pointed out: *"In addition, doing a continuous number of hours with all class every day would, in my opinion, be quite harmful to the eyes, especially considering that electronic devices are then also used for other activities (texting, calls, research, movies, social media...)"*

#### Quality of lessons

Distance education may facilitate the management of the class group, leading to some advantages such as better time management of the lesson and leading to the perception of being heard more by the class group (humanities teachers: 36 references, 39%; primary teachers: 22 references, 24%; science teacher: 18 references, 20%; preservice teachers: 16 references, 17%). In addition, the possibility of using tools not easily accessible in the classroom, such as sharing platforms, improves students’ autonomy and encourages collaborative work. Indeed, as one upper humanities teacher wrote: *“Kids don't chat with each other. I can explain in peace because students don't interrupt. If the students get distracted, I don't notice or get upset.” Similarly, one primary teacher exclaimed: "There is an incredible speed of information exchange, making students more creative and autonomous”. Furthermore, one upper science teacher said: “There are no discipline problems, those who are interested are more involved, and more time is given to the student to assimilate concepts”.*

Some teachers also reported problems due to a lack of student concentration during the lessons. At home, students may be less focused because they have more distractions (preservice teachers: 32 references, 28%; humanities teachers: 32 references, 28%; primary teachers: 27; 24%; science teachers: 22 references, 20%). Teachers reported that students often kept their cameras off. For instance, one secondary school teacher of humanities explained: *“There is a lack of continuous and stimulating feedback from pupils; their contribution to learning during the lesson is often crucial”.* The problem became particularly salient when it was time for evaluation as one science teacher reported: *“Great limitations are the assessment and evaluation: it is impossible to check if the tests are carried out regularly, with agreed instruments (for example for students with special education needs), or not. Often students rely more on the search for a ploy than on study and on their own abilities (they are kids)”*.

Finally, it is worth noting that 25 teachers reported finding no benefits in distance education when asked to indicate what positive aspects were related to the use of distance teaching, meaning that in their opinion distance education cannot be a useful alternative to classroom teaching in any way. This view was not characteristic of teachers in any grade, or subject area.

## Discussion

The sudden spread of COVID-19 that resulted in school closures posed questions and concerns about the relationship between teaching and technology. In this respect, the present mixed method study explored this relationship. On the one hand, we evaluated factors predisposing the use of technology and how it varied according to teachers’ school grade level and teaching subject. On the other hand, the opinions and thoughts of participants regarding the positive and negative aspects of distance teaching allowed us to enrich our understanding and the value of the quantitative data.

Concerning quantitative results, we found that preservice teachers showed a greater level of self-efficacy compared to humanities teachers while primary teachers presented a greater level than both science and humanities teachers. With regard to preservice teachers, they were likely to have been influenced by their previous ‘apprenticeship of observation’ model (Lortie, 2020), which is drawn from experiences of their twelve years of schooling, causing them to believe that they were already capable teachers (Pendergast et al., 2011). Another consideration is that some participants could also be parents and may have been influenced by observations of their own children’s schooling (Pendergast et al., 2011). Besides, another possible explanation concerns the long period of teaching internship that Italian students had already carried out under the supervision of a senior teacher. Therefore, it is possible that this previous experience in which preservice teachers watched a senior teacher give instructional practices positively affected their perceived self-efficacy in handling classroom situations (Dassa & Nichols, 2019). For these reasons, a direct link between higher teaching self-efficacy and subsequent competence in classroom practice should not be assumed (Gravett et al., 2011). Instead, this measure represents only preservice teachers’ perception of confidence in teaching and their own abilities.

Regarding primary teachers, the literature on self-efficacy among different teachers’ grade levels is scarce, and our results suggest the need to improve research on this issue. Betoret (2006), for example, found that self-efficacy was slightly higher for primary teachers than secondary teachers, but it did not reach statistical significance. Stephanou and Oikonomou (2018), despite no differences in self-efficacy, found that primary school teachers had a significantly stronger sense of school collective efficacy compared to secondary school teachers. Although it is a gap in the literature, we feel that this difference may be affected by different academic paths. Indeed, in Italy the master’s degree to become a primary teacher includes several courses on psychological and pedagogical issues. By contrast, Italian teachers in secondary schools have obtained a master's degree in their subject specialization. Thus, we surmise that less attention to psychology and pedagogy could affect their teachers’ self-efficacy, making secondary school teachers less confident in their teaching.

However, when we assessed online teaching self-efficacy, no differences between primary and secondary teachers emerged. This finding should not surprise us. Indeed, distance teaching not only involves a transfer of knowledge from the classroom environment to the virtual one, but it includes broader and different challenges than those involved in traditional face-to-face teaching (Horvitz et al., 2015). Besides, the implementation of distance education met specific difficulties in Italy due to the lack of specific information on its management and the developed technological infrastructure as well, factors which are present regardless of teachers’ grade level or their subject area (Giovannella et al., 2020, Pellegrini & Maltinti, 2020). Moreover, as revealed by OECD data (2018), half of the Italian teachers had not received formal training in using technology for teaching purposes before the spread of COVID-19. Many of them reported not feeling prepared to use it. A lack of previous training for teachers could compromise online teaching self-efficacy, affecting the quality of distance education.

Regarding the facilitating conditions for distance education and the perceived ease of use of technology, significant differences between teacher groups emerged from our study. We found that primary teachers showed a lower level of facilitating conditions for distance education than humanities and science teachers and of the perceived ease of use of technology compared to science teachers. However, we surmise that our research may be rapresentative of the Italian context, in which the use of technology in primary schools is only a recent development (Oddone, 2016). Therefore, it is likely that secondary teachers (both humanities and science subjects) may feel more supported in distance education, perceiving a greater level of facilitating conditions. Moreover, primary school teachers may also have had less experience in using technology and this may have affected their perception of the ease of use of technology which reached a significant difference compared to science teachers who could have had more previous experience using technology (Baki et al., 2018). Furthermore, our findings would suggest that the greater self-efficacy of primary teachers, but no difference in online self-efficacy when compared to secondary teachers, could suggest that primary teachers were willing, but were perhaps limited in their capacity to easily implement distance education.

For what about technology skills, we found a significant group effect concerning basic technology skills, even if differences among groups did not reach a significant level. We assume that simple skills (e.g., using word sheets) would have already been attained by most teachers, and would have reached a ceiling effect. By contrast, in terms of advanced technology skills (such as the ability to create and/or modify web pages), or technologies used for education, a significantly lower level of skill was found among teachers. Indeed, humanities teachers showed a lower level of advanced technology skills compared to other groups and of technology for pedagogy compared to science teachers. We think that these results could be explained by considering the pedagogical beliefs of teachers in relation to their subjects. Indeed, as previously discussed, humanities teachers are likely to perceive their subjects as “human-focused nature” (John & La Velle, 2004). For these teachers, communication is essential and the teacher-student connectedness is enhanced using facial expressions and body language (Bao, 2020). On the contrary, science teachers held relatively open attitudes towards the potential of technology to transform teaching, in line with the role of mathematics in the evolution of digital technologies (John & La Velle, 2004). Thus, the forced shift in digital learning may have led to the difficulty, especially for humanities teachers, in their ability to communicate effectively with students and restricted them from generalizing the teaching ability developed in the physical classroom into online contexts, affecting both the advanced technology skills as well as the technology for pedagogy (Putra et al., 2020). Noteworthy is that, Yang and colleagues (2020) found a similar result showing that 26-35 years old science teachers of primary and secondary schools had a higher acceptance rate to technology than the 26-35 years liberal arts teachers. Authors hypothesized that this difference could be due to different levels of skills included in the “Technological Pedagogical Content Knowledge theory” (TPACK, Koehler & Mishra, 2009). Overall, TPACK represents a dynamic framework describing the pedagogical and content knowledge that teachers must rely on to implement curriculum and instruction with digital technologies (Koehler & Mishra, 2009; Schmidt et al., 2009). Yang and colleagues (2020) stated that science teachers could have a greater level of technology knowledge and pedagogical content knowledge. In other words, it was easier for science teachers to integrate their pedagogical knowledge into technological tools. Tokmak et al. (2013) found that these differences were already present among preservice teachers: preservice math teachers significantly presented more excellent technology knowledge and technological content knowledge than social science teachers.

One of the critical drivers to integrating technology in teaching, the behavioural intention to use technology, was also examined in our study. We found that preservice teachers had a significantly higher level of intention to use technology compared to primary teachers, humanities, and science teachers. This finding is aligned with previous research in which preservice teachers reported strong positive beliefs in technology and a solid readiness to use technology in the classroom (Farjon et al., 2019; Okumuş & Yurdakal, 2016; Şad and Göktaş, 2014). Indeed, it is likely that many preservice teachers are part of the net generation that already actively use technology in everyday living. Furthermore, many preservice teacher education programmes make specific reference to technology, which is increasingly seen as a mandatory component of teacher accreditation (McGarr & Gavaldon, 2018). With respect to the Italian context, although technology is not a mandatory component of curricula, a substantial effort to integrate technology in different ways was being made before the spread of COVID-19 (Tarchi et al., 2022) showing that, generally, Italian preservice teachers showed great digital readiness (Tarchi et al., 2022).

For what about the qualitative findings, the first theme was related to the use of technology. In its positive meaning, many teachers appreciated the possibility of increasing their technological knowledge for professional development, considering it as a fundamental part to implement, both for students and teachers. This finding was also mirrored by a qualitative study on a sample of Italian high school students (Tzankova et al., 2022). Indeed, many students reported that technology could be used in multiple ways and could be part of learning beyond social networks. Moreover, students considered the availability of computers and the internet as the only opportunity to continue schooling in the COVID-19 emergency. In its negative meaning, teachers and preservice teachers in our study reported many challenges such as technical problems and the fear that the technology could fuel social differences among students, unfortunately, not being wrong. Indeed, as shown by the Italian National Institute of Statistics (ISTAT; www.istat.it), 45% of youths (age 6 to 17) struggled to cope with distance education due to a lack of devices in their homes, increasing the risk of school disruption. Similar concerns were also found at the international level, as suggested by several studies (Atmojo and Nugroho, 2020; Bergdahl and Nouri, 2021; Devkota, 2021).

Besides, looking at our sample differences, it is worth noting that the use of technology to create pedagogical content is mentioned mainly by preservice and primary school teachers. By contrast, secondary school teachers, mainly from humanities subjects, seem to place more importance on factors related to assessment and lesson quality. Similar findings regarding the use of technology in implementing pedagogical content among Italian teachers were found even before COVID-19 (Legrottaglie & Ligorio 2014). Indeed, they showed that Italian upper secondary teachers referred to technology as being associated with the didactic dimension and with teaching-learning procedures. By contrast, primary school teachers referred to the dimension of technologies as being capable of creating playful moments.

The social relationship with students was another critical and cross-cutting theme for Italian teachers. While many teachers appreciated the possibility of continuing to see their students, many complained about the lack of a real relationship, for which technological devices cannot compensate. Our findings in this respect are aligned with many other international studies. For example, Niemi and Kousa (2020) found that Finnish teachers positively valued the opportunity to continue to see their students but, at the same time, they had difficulty creating real interactive relationships with students, feeling that the interaction was too artificial. Hebebhci and colleagues (2020) found similar results among Turkish teachers. When interviewed, many teachers claimed that not being in the same physical environment limited the interaction and that online courses could not replace regular lessons. Carrillo and Flores (2020), in their literature review about online teaching and learning practices, noted that the social presence in distance teaching was a topic included in most of the literature related to distance teaching even before the spread of COVID-19. In the Italian context, Addimando et al. (2021) reported that most primary teachers were troubled by the lack of physical contact, considered fundamental for students at that age. By contrast, although concerned about the lack of face-to-face lessons, Italian university teachers reported an appreciation that technology allowed students and teachers to have more continuous contact outside the lesson time, unthinkable before the lockdown (Casacchia et al., 2021). Italian students reported similar mixed feelings: they experienced inhibited relationships with peers and teachers, but, on the other hand, the situation allowed adolescents to look at their relationships with teachers and classmates in a different and new way rather than take them for granted (Tzankova et al., 2022).

The third thematic area was related to the versatility of distance education since it allows everyone to attend, without the need to be physically present. Indeed, many teachers of the present study reported how the versatility of distance education represented an attractive feature as it can allow students and teachers to manage their teaching/learning time and location according to their specific needs. The versatility of distance education is recognized as a key element by a great deal of the literature, even before the COVID-19 pandemic (Bates, 2005). In this regard, Italian students had similar views: they appreciated the flexibility of distance education, choosing what to study within a subject; how and when to study, and the possibility of using asynchronous activities and materials (Tzankova et al., 2022). Moreover, as reported by teachers in the study, the versatility of distance education allowed them to save time (and therefore get up later in the morning) and not crowd public transportation. However, disadvantages such as increased workload and health hazards were also suggested and they are in line with many other studies both at the national and international level (Casacchia et al., 2021; Kaden, 2020; Niemi & Kousa, 2020; Oliveira et al., 2021). Finally, it is interesting to note that Italian teachers, and in particular teachers of the humanities, seemed to have been very worried about their and students’ physical health.

The quality of lessons was the last theme that emerged from the qualitative data. Teachers suggested that distance lessons facilitated the class management, especially for humanities teachers. Moreover, the use of technological tools allowed for more interactive and creative lessons. Interestingly, this aspect was very important for Italian students (Tzankova et al., 2022). Indeed, students appreciated teachers’ efforts to use online tools creatively. However, students stated that many of them just continued to teach traditionally without exploiting the full potential of the technology (Tzankova et al., 2022). This opinion aligns with another national survey (Mascheroni et al., 2021) of students aged between 14 and 18 that reported that 44% of them said that their teachers simply transferred the traditional lessons onto the computer screen while just a few of them introduced innovations in their lessons to utilise technological means better.

Another feature was the challenge of evaluating students’ attentiveness and actual preparation. Indeed, teachers expressed concern about the difficulty of understanding their students' real attention and receiving immediate feedback, especially for secondary school teachers. This view is supported by a national survey conducted on students (Mascheroni et al., 2021), which showed that distance education made it more difficult for 7 out of 10 students to concentrate during distance lessons. Moreover, this finding aligns with other qualitative and quantitative studies among teachers, both national and international, which have highlighted the difficulties in monitoring students during classes, the possibility of cheating during exams, and worries about a possible decrease in learning efficiency (Alqurshi, 2020; Casacchia et al., 2021; Niemi & Kousa, 2020; Süğümlü, 2021).

To sum up, we employed a mixed-method study to triangulate findings to offset some limitations within each of the respective methods (Creamer, 2018). The quantitative findings shed light on factors that can increase teacher readiness to switch to online environments. In this regard, our results revealed that preservice teachers showed good levels of self-efficacy and seemed particularly willing to integrate technology into their lessons, as demonstrated by the high level of behavioural intention to use technology. Primary teachers reported a high level of self-efficacy too. Still, they perceive themselves as not adequately supported in integrating and using technology due to the scarce facilitating conditions and low level of perceived ease of use. By contrast, secondary school teachers perceived several facilitating conditions in integrating technology. However, differences concerning subjects emerged. Indeed, humanities teachers reported more limited technological competencies, as shown by the low levels of advanced technology skills and technology for pedagogy. By contrast, science teachers seemed to perceive themselves as more confident in the use of technology with great levels in the aforementioned variables and the perceived ease of use of technology. On the other hand, qualitative results allowed us to investigate what positive and negative aspects teachers encountered during distance teaching. From the analysis of their answers, the lack of relationship was the main transversal theme with respect to grade levels or subject areas. The use of technology, both in its positive and negative meaning, was cited especially by preservice teachers and primary teachers. Concerning the versatility of distance education, it appeared to be present above all among preservice teachers and humanities teachers, while aspects of lesson quality were present above all among secondary school teachers and less among primary and preservice teachers. To conclude, teachers’ opinions represent a crucial added value to the knowledge of those factors on which institutions and policy may focus, to improve the quality of didactic teaching and promote best practices during these extraordinary conditions

### Limitations

The first study limitation concerns the demographic composition of the sample as the number of females was greater than males, even if in line with the actual Italian context where 78% of teachers are women (OCSE TALIS, 2018). Further research should analyze the role of gender since contrasting results have been reported. For example, in some studies females reported less use of technology than males (Saleh Mahdi & Al-Dera, 2013; Teo et al., 2015), while in other studies no gender differences were described among teachers (Wong & Hanafi, 2007). The second limitation concerns the geographical distribution of the sample because most of our teachers came from Northern Italy. Although Italy has a centralized school system, some differences between the North and South could have affected our findings (Ballarino et al., 2014) Conversely, a more balanced data collection between Northern and Southern Italy would boost the generalization of our findings.. The third limitation implies the low number of teachers from low upper secondary schools in our sample. Further research including this sample would provide insight into how teachers see technology for teaching with pre-adolescent students. The fourth limitation was the qualitative part of the study, which was based on the analysis of open-ended questions. In depth-interviews would certainly have offered a broader view of teachers’ personal opinions, shedding further light on relevant details and possible contradictions hidden behind open-ended questions. Another limitation of our study is the lack of information about the days already spent by teachers in distance education. We can speculate that a different experience with distance education can affect both factors promoting technologies and experiences. Adding this variable in future studies could support the understanding of results and their generalizations. At the end, we need to be careful in the interpretation from findings collected with preservice teachers. Indeed we believe that their opinions and feelings on distance education were important to understand how implement distance education, even if they are hypothetical and not based on their direct experience.

### Further Research and Practical Implications

The present study should be considered as a starting point for further analysis, research, and surveys. First of all, it would be of interest to other educational jurisdictions to compare the perspective related to opinions and personal factors of Italian school teachers with teachers in other countries. Indeed, although it is undeniable that COVID-19 affected the whole world, some countries were more impacted by the pandemic than others, where different lengths of distance e-learning occurred. For example, in South Australia distance education was in place for a total of 5 weeks among primary and secondary schools, while schools in Arizona, USA, utilized distance learning for almost the entirety of the 2-year pandemic period prior to the 2021-22 academic year. Moreover, some countries, especially in Northern Europe, have a long tradition of using technology in teaching while others, like Italy, still struggle. Therefore, it would be worthwhile to understand if the personal factors investigated in the present study would differ among countries, considering the policy and efforts involved in the integration of technology in schools. Indeed, as mentioned before, Italian schools had a long period of distance education, moreover digitization in Italy is still behind compared to other European countries. For example the index DESI 2018 (Digital Economy and Society Index), through which the European Commission measures the level of implementation of the Digital Agenda in the different member states, depicted a situation whereby Italy ranks 25th out of 28 countries. Moreover, surveys on pedagogical innovation and teachers' professional development (OECD PISA, 2010; OECD, 2015) have shown that Italy has been behind most European countries concerning equipment and usage of technologies in school (Calvani, 2013). For these reasons, we believe that Italian results could be generalized to those countries which similarly had a long period of distance education and previous difficulties in integrating technology. Another interesting comparison would be between Italian school teachers and university teachers and between school teachers, students and parents. Indeed, teachers and students shared common views about distance education, its benefits, and its difficulties, as discussed above. However, comparing the perspectives of all stakeholders should be carried out via local case studies, allowing deep comparison between individuals that belong to the same context.

To conclude, distance education has been a necessary means to allow students to continue their education and keep the relationship with classmates and teachers alive, albeit behind a screen. However, the limitations that have emerged are many, both due to contextual factors, such as the lack of accessibility and internet connection, the lack of central coordination in Italy and of teachers’ technological expertise and due to the intrinsic nature of distance education, which does not allow interaction and exchange, especially for the youngest of students. Since we cannot exclude the need to use distance education in the future, it is crucial to be ready to implement distance education learning from our experience, improving possibilities and reducing risks. It could be helpful, for example, to provide different training based on school order and subject taught. From this view, it would be essential to reinforce the technology for pedagogy, especially among secondary school teachers of humanities. In contrast, primary school teachers could more enjoy training focused on increasing the perceived ease of use of technology. Furthermore, we feel that some practical suggestions could be derived from teachers’ opinions and experiences: teachers could try to provide a balance of asynchronous and synchronous lessons trying to exploit the versatility of distance education; choose tools that are mobile-friendly and/or can be used offline also to benefit those students who do not have constant access to the network; to promote group work and ensuring students’ understanding with mini-assessments. Of course, it is necessary to guarantee that educators and students have infrastructure and hardware (i.e., computers, tablets etc) to participate in distance education, especially those thare are more vulnerable. The last important consideration regards technology as a support tool in face-to-face teaching. Indeed, already in 2016, the European Commission had encouraged member states to foster the development of a new digitalised learning environment. In addition, several studies have reported that the integration of technology into instruction is an essential ingredient for student success in the 21st-century (Foster et al., 2011; Harter, 2011; Washbon, 2012). In light of this, UNESCO ICT Competency Framework for teachers emphasized the urgent need for teachers to gain knowledge, skills, and attitudes required to integrate modern tools and resources into the learning process (Oddone, 2016). Therefore, it is important to create and implement initiatives and training to encourage appropriate technology among classroom teachers. Indeed, what happened could represent a further stimulus to rethink technology in teaching in a more integrated and informed way.

## References

[CR1] Abbasi MS, Chandio FH, Soomro AF, Shah F (2011). Social influence, voluntariness, experience and the internet acceptance: An extension of technology acceptance model within a south-Asian country context. Journal of Enterprise Information Management.

[CR2] Abdullah F, Ward R (2016). Developing a General Extended Technology Acceptance Model for E-Learning (GETAMEL) by analysing commonly used external factors. Computers in Human Behavior.

[CR3] Addimando L, Leder D, Zudini V (2021). Teaching and Learning in the COVID-19 Era: The Experience of an Italian Primary School Class. Turkish Online Journal of Educational Technology-TOJET.

[CR4] Al-Awidi HM, Alghazo IM (2012). The effect of student teaching experience on preservice elementary teachers’ self-efficacy beliefs for technology integration in the UAE. Education Tech Research Dev.

[CR5] Alea LA, Fabrea MF, Farooqi AZ, Roldan RDA (2020). Teachers’ Covid-19 awareness, distance learning education experiences and perceptions towards institutional readiness and challenges. International Journal of Learning, Teaching and Educational Research.

[CR6] Alhumaid K, Ali S, Waheed A, Zahid E, Habes M (2020). COVID-19 &Elearning: Perceptions &Attitudes Of Teachers Towards E-Learning Acceptance in The Developing Countries. Multicultural Education.

[CR7] Alqurshi A (2020). Investigating the impact of COVID-19 lockdown on pharmaceutical education in Saudi Arabia – A call for a remote teaching contingency strategy. Saudi Pharmaceutical Journal.

[CR8] Anderson E, Hira A (2020). Loss of brick-and-mortar schooling: How elementary educators respond. Information and Learning Sciences.

[CR9] Anderson SE, Groulx JG, Maninger RM (2011). Relationships among Preservice Teachers’ Technology-Related Abilities, Beliefs, and Intentions to Use Technology in Their Future Classrooms. Journal of Educational Computing Research.

[CR10] Antonietti A, Giorgetti M (2006). Teachers’ beliefs about learning from multimedia. Computers in Human Behavior.

[CR11] Armenteros M, Liaw S-S, Fernández M, Díaz RF, Sánchez RA (2013). Surveying FIFA instructors’ behavioral intention toward the Multimedia Teaching Materials. Computers & Education.

[CR12] Atmojo AEP, Nugroho A (2020). EFL Classes Must Go Online! Teaching Activities and Challenges during COVID-19 Pandemic in Indonesia. Register Journal.

[CR13] Baki, R., Birgoren, B., & Aktepe, A. (2018). A Meta Analysis of Factors Affecting Perceived Usefulness and Perceived Ease of Use in The Adoption of E-Learning Systems. *Turkish Online Journal of Distance Education*, 4–42. 10.17718/tojde.471649

[CR14] Ballarino G, Panichella N, Triventi M (2014). School expansion and uneven modernization. Comparing educational inequality in Northern and Southern Italy. Research in Social Stratification and Mobility.

[CR15] Banas, J. R., & York, C. S. (2014). Authentic learning exercises as a means to influence preservice teachers’ technology integration self-efficacy and intentions to integrate technology. *Australasian Journal of Educational Technology, 30*(6). 10.14742/ajet.362

[CR16] Bao W (2020). COVID-19 and online teaching in higher education: A case study of Peking University. Human Behavior and Emerging Technologies.

[CR17] Bates AT (2005). *Technology, e-learning and distance education*.

[CR18] Bergdahl N, Nouri J (2021). Covid-19 and Crisis-Prompted Distance Education in Sweden. Tech Know Learn.

[CR19] Betoret FD (2006). Stressors, Self-Efficacy, Coping Resources, and Burnout among Secondary School Teachers in Spain. Educational Psychology.

[CR20] Brouwers A, Tomic W (2003). A Test of the Factorial Validity of the Teacher Efficacy Scale. Research in Education.

[CR21] Calvani A (2013). Why introduce ICT in schools? A road map for decision makers and teachers. Italian Journal of Educational Technology.

[CR22] Cardullo V, Wang C, Burton M, Dong J (2021). K-12 teachers’ remote teaching self-efficacy during the pandemic. Journal of Research in Innovative Teaching & Learning.

[CR23] Carrillo C, Flores MA (2020). COVID-19 and teacher education: A literature review of online teaching and learning practices. European Journal of Teacher Education.

[CR24] Casacchia M, Cifone MG, Giusti L (2021). Distance education during COVID 19: an Italian survey on the university teachers’ perspectives and their emotional conditions. BMC Med Educ.

[CR25] Cataudella S, Carta SM, Mascia ML, Masala C, Petretto DR, Agus M, Penna MP (2021). Teaching in Times of the COVID-19 Pandemic: A Pilot Study on Teachers’ Self-Esteem and Self-Efficacy in an Italian Sample. International Journal of Environmental Research and Public Health.

[CR26] Chang C-T, Hajiyev J, Su C-R (2017). Examining the students’ behavioral intention to use e-learning in Azerbaijan? The General Extended Technology Acceptance Model for E-learning approach. Computers & Education.

[CR27] Chen YJ, Chien HM, Kao CP (2019). Online searching behaviours of preschool teachers: A comparison of pre-service and in-service teachers’ evaluation standards and searching strategies. Asia-Pacific Journal of Teacher Education.

[CR28] Christophersen KA, Elstad E, Turmo A, Solhaug T (2016). Teacher education programmes and their contribution to student teacher efficacy in classroom management and pupil engagement. Scandinavian Journal of Educational Research.

[CR29] Cordes, C., & Miller, E. (2000). *Fool’s gold: A critical look at computers in childhood.*

[CR30] Creamer EG (2018). Enlarging the Conceptualization of Mixed Method Approaches to Grounded Theory With Intervention Research. American Behavioral Scientist.

[CR31] Crowe S, Howard AF, Vanderspank-Wright B, Gillis P, McLeod F, Penner C, Haljan G (2021). The effect of COVID-19 pandemic on the mental health of Canadian critical care nurses providing patient care during the early phase pandemic: A mixed method study. Intensive and Critical Care Nursing.

[CR32] Danchikov, E. A., Prodanova, N. A., Kovalenko, Y. N., & Bondarenko, T. G. (2021). Using different approaches to organizing distance learning during the COVID-19 pandemic: Opportunities and disadvantages. *Linguistics and Culture Review*, *5*(S1), 587–595. 10.21744/lingcure.v5nS1.1444

[CR33] Dassa L, Nichols B (2019). Self-Efficacy or Overconfidence? Comparing Preservice Teacher Self-Perceptions of Their Content Knowledge and Teaching Abilities to the Perceptions of Their Supervisors. The New Educator.

[CR34] Davis FD, Bagozzi RP, Warshaw PR (1989). User acceptance of computer technology: A comparison of two theoretical models. Management Science.

[CR35] Devkota KR (2021). Inequalities reinforced through online and distance education in the age of COVID-19: The case of higher education in Nepal. International Review of Education.

[CR36] Elo S, Kyngäs H (2008). The qualitative content analysis process. Journal of Advanced Nursing.

[CR37] Ertmer, P. A., & Ottenbreit-Leftwich, A. T. (2010). Teacher technology change: How knowledge, confidence, beliefs, and cultureintersect. *Journal of Research on Technology in Education,**42*(3), 255–284. 10.1080/15391523.2010.10782551

[CR38] Ertmer, P. A., Ottenbreit-Leftwich, A. T., & Tondeur, J. (2015). Teacher beliefs and uses of technology to support 21st century teaching and learning. *Handbook of Research on Teachers’ Beliefs.* Routledge.

[CR39] European Commission. (2016). Digital Single Market. Digital Economy &Society. Available at https://digital-strategy.ec.europa.eu/en/library/desi-2016-country-profiles-slides.

[CR40] Farjon D, Smits A, Voogt J (2019). Technology integration of pre-service teachers explained by attitudes and beliefs, competency, access, and experience. Computers & Education.

[CR41] Foster J, Kelley P, Pritz S, Hodes C (2011). CTE’s Focus on Continuous Improvement. Techniques: Connecting Education and Careers (J1).

[CR42] Giovannella C, Passarelli M, Persico D (2020). The Effects of the Covid-19 Pandemic on Italian Learning Ecosystems: The School Teachers’ Perspective at the steady state. Interaction Design and Architecture(s) Journal.

[CR43] Goodson IF, Mangan JM (1995). Subject Cultures and the Introduction of Classroom Computers. British Educational Research Journal.

[CR44] Gravett S, Henning E, Eiselen R (2011). New teachers look back on their university education: Prepared for teaching, but not for life in the classroom. Education as Change.

[CR45] Hammond M, Reynolds L, Ingram J (2011). How and why do student teachers use ICT?. Journal of Computer Assisted Learning.

[CR46] Harter C (2011). Making Connections: Integrating Computer Applications with the Academic Core. Techniques: Connecting Education and Careers (J1).

[CR47] Hatlevik OE (2017). Examining the Relationship between Teachers’ Self-Efficacy, their Digital Competence, Strategies to Evaluate Information, and use of ICT at School. Scandinavian Journal of Educational Research.

[CR48] Hebebci MT, Bertiz Y, Alan S (2020). Investigation of views of students and teachers on distance education practices during the Coronavirus (COVID-19) Pandemic. International Journal of Technology in Education and Science.

[CR49] Hennessy S, Ruthven K, Brindley S (2005). Teacher perspectives on integrating ICT into subject teaching: Commitment, constraints, caution, and change. Journal of Curriculum Studies.

[CR50] Henson, R. K. (2011). *Teacher Self-efficacy: Substantive Implications and Measurement Dilemmas* [E Educational Research Exchange]. e Educational Research Exchange, Texas A&M University. https://files.eric.ed.gov/fulltext/ED452208.pdf

[CR51] Hermans R, Tondeur J, van Braak J, Valcke M (2008). The impact of primary school teachers’ educational beliefs on the classroom use of computers. Computers & Education.

[CR52] Horvitz, B. S., Beach, A. L., Anderson, M. L., et al. (2015). Examination of faculty self-efficacy related to online teaching. *Innovative Higher Education,**40,* 305–316. 10.1007/s10755-014-9316-1

[CR53] Howard, S., & Maton, K. (2011). Theorising knowledge practices: A missing piece of the educational technology puzzle. *Research in Learning Technology, 19*(3). 10.3402/rlt.v19i3.17109

[CR54] Hu PJ-H, Clark TH, Ma WW (2003). Examining technology acceptance by school teachers: A longitudinal study. Information & Management.

[CR55] Hughes JE, Cheah YH, Shi Y, Hsiao KH (2020). Preservice and inservice teachers' pedagogical reasoning underlying their most-valued technology-supported instructional activities. Journal of Computer Assisted Learning.

[CR56] Hurmerinta-Peltomäki L, Nummela N (2006). Mixed methods in international business research: A value-added perspective. Management International Review.

[CR57] Hussein MH, Hock Ow S, Ibrahin I, Mahmoud MA (2021). Measuring instructors continued intention to reuse Google Classroom in Iraq: A mixedmethod study during COVID-19. Interactive Technology and Smart Education.

[CR58] Ismailos L, Gallagher T, Bennett S, Li X (2022). Pre-service and in-service teachers’ attitudes and self-efficacy beliefs with regards to inclusive education. International Journal of Inclusive Education.

[CR59] John PD, La Velle LB (2004). Devices and desires: Subject subcultures, pedagogical identity and the challenge of information and communications technology. Technology, Pedagogy and Education.

[CR60] Kaden U (2020). COVID-19 School Closure-Related Changes to the Professional Life of a K–12 Teacher. Education Sciences.

[CR61] Karaseva, A., Siibak, A., & Pruulmann-Vengerfeldt, P. (2015). Relationships between teachers` pedagogical beliefs, subject cultures, and mediation practices of students’ use of digital technology. *Cyberpsychology: Journal of Psychosocial Research on Cyberspace, 9*(1). 10.5817/CP2015-1-6

[CR62] Kebritchi M, Lipschuetz A, Santiague L (2017). Issues and Challenges for Teaching Successful Online Courses in Higher Education: A Literature Review. Journal of Educational Technology Systems.

[CR63] Koc M, Gulyagci S (2013). Facebook Addiction Among Turkish College Students: The Role of Psychological Health, Demographic, and Usage Characteristics. Cyberpsychology, Behavior, and Social Networking.

[CR64] Koçoglu E, Tekdal D (2020). Analysis of Distance Education Activities Conducted during COVID-19 Pandemic. Educational Research and Reviews.

[CR65] Koehler M, Mishra P (2009). What is technological pedagogical content knowledge (TPACK)?. Contemporary Issues in Technology and Teacher Education.

[CR66] König J, Jäger-Biela DJ, Glutsch N (2020). Adapting to online teaching during COVID-19 school closure: Teacher education and teacher competence effects among early career teachers in Germany. European Journal of Teacher Education.

[CR67] Korkmaz G, Toraman Ç (2020). Are we ready for the post-COVID-19 educational practice? An investigation into what educators think as to online learning. International Journal of Technology in Education and Science.

[CR68] Košir, K., Dugonik, Š., Huskić, A., Gračner, J., Kokol, Z., & Krajnc, Ž. (2020). Predictors of perceived teachers’ and school counsellors’ work stress in the transition period of online education in schools during the COVID-19 pandemic. *Educational Studies*, 1–5. 10.1080/03055698.2020.1833840

[CR69] Krumsvik RJ (2014). Teacher educators’ digital competence. Scandinavian Journal of Educational Research.

[CR70] Lee, Y.-L., Chen, H.-C., & Chan, Y.-C. (2019). The attentional bias of gelotophobes towards emotion words containing the Chinese character for ‘laugh’: An eye-tracking approach. *Current Psychology*, 1–14.

[CR71] Legrottaglie, S., & Ligorio, M. B. (2014). L’uso delle tecnologie a scuola: Il punto di vista dei docenti. *Italian Journal of Educational Technology, 22*(3). 10.17471/2499-4324/188

[CR72] Lortie DC (2020). *Schoolteacher: A sociological study*.

[CR73] Ma, K., Chutiyami, M., Zhang, Y., & Nicoll, S. (2021). Online teaching self-efficacy during COVID-19: Changes, its associated factors and moderators. *Education and Information Technologies.*10.1007/s10639-021-10486-3 10.1007/s10639-021-10486-3 PMC7946405 33723481

[CR74] Magen-Nagar N, Firstater E (2019). The Obstacles to ICT Implementation in the Kindergarten Environment: Kindergarten Teachers’ Beliefs. Journal of Research in Childhood Education.

[CR75] Mailizar, M., Burg, D., & Maulina, S. (2021). Examining university students’ behavioural intention to use e-learning during the COVID-19 pandemic: An extended TAM model. *Education and Information Technologies.*10.1007/s10639-021-10557-5 10.1007/s10639-021-10557-5 PMC8079853 33935579

[CR76] Mascheroni G., Saeed M., Valenza M., Cino D., Dreesen T., Zaffaroni L.G., Winther K., UNICEF Office of Research - Innocenti, 2021. *"Learning at a Distance: Children’s remote learning experiences in Italy during the COVID-19 pandemic*," Innocenti Research Report.

[CR77] McGarr O, Gavaldon G (2018). Exploring Spanish pre-service teachers’ talk in relation to ICT: Balancing different expectations between the university and practicum school. Technology, Pedagogy and Education.

[CR78] Menabò, L., Sansavini, A., Brighi, A., Skrzypiec, G., & Guarini, A. (2021). Promoting the integration of technology in teaching: An analysis of the factors that increase the intention to use technologies among Italian teachers. *Journal of Computer Assisted Learning*, jcal.12554. 10.1111/jcal.12554

[CR79] Niemi HM, Kousa P (2020). A Case Study of Students’ and Teachers’ Perceptions in a Finnish High School during the COVID Pandemic. International Journal of Technology in Education and Science.

[CR80] Oddone, F. (2016). Cloud Computing Applications and Services fostering Teachers’ Self-efficacy. *Journal of E-Learning and Knowledge Society*, *12*(2). https://www.learntechlib.org/p/173463/

[CR81] OECD. (2015). Digital economy outlook 2015. Directorate for Science, Technology and Innovation, OECD.

[CR82] OECD Publishing (2010). PISA PISA 2009 results: What makes a school successful?: Resources, Resources, Policies and Practices (Volume IV).

[CR83] Okumuş, K., & Yurdakal, İ. H. (2016). Peer feedback through snss social networking sites pre service teachers views about using facebook for peer feedback on microteachings. *Elementary Education Online 15*(4), 1206–1216. 10.17051/io.2016.17666

[CR84] Oliveira G, Grenha Teixeira J, Torres A, Morais C (2021). An exploratory study on the emergency remote education experience of higher education students and teachers during the COVID-19 pandemic. British Journal of Educational Technology.

[CR85] Östlund U, Kidd L, Wengström Y, Rowa-Dewar N (2011). Combining qualitative and quantitative research within mixed method research designs: A methodological review. International Journal of Nursing Studies.

[CR86] Pajares MF (1992). Teachers’ Beliefs and Educational Research: Cleaning Up a Messy Construct. Review of Educational Research.

[CR87] Pellegrini M, Maltinti C (2020). ‘School Never Stops’: Measures and Experience in Italian Schools during the COVID-19 Lockdown. Best Evid Chin Edu.

[CR88] Pellerone M (2021). Self-Perceived Instructional Competence, Self-Efficacy and Burnout during the Covid-19 Pandemic: A Study of a Group of Italian School Teachers. European Journal of Investigation in Health, Psychology and Education.

[CR89] Pendergast, D., Garvis, S., & Keogh, J. (2011). Pre-Service Student-Teacher Self-efficacy Beliefs: An Insight Into the Making of Teachers. *Australian*. *Journal of Teacher Education, 36*(12). 10.14221/ajte.2011v36n12.6

[CR90] Popa D, Repanovici A, Lupu D, Norel M, Coman C (2020). Using Mixed Methods to Understand Teaching and Learning in COVID 19 Times. Sustainability.

[CR91] Portillo J, Garay U, Tejada E, Bilbao N (2020). Self-Perception of the Digital Competence of Educators during the COVID-19 Pandemic: A Cross-Analysis of Different Educational Stages. Sustainability.

[CR92] Pressley T, Ha C (2021). Teaching during a Pandemic: United States Teachers’ Self-Efficacy During COVID-19. Teaching and Teacher Education.

[CR93] Putra, P., Liriwati, F. Y., Tahrim, T., Syafrudin, S., & Aslan, A. (2020). The Students Learning from Home Experiences during Covid-19 School Closures Policy In Indonesia. *Jurnal Iqra’ : Kajian Ilmu Pendidikan*, *5*(2), 30–42. 10.25217/ji.v5i2.1019

[CR94] Rahayu RP, Wirza Y (2020). Teachers’ Perception of Online Learning during Pandemic Covid-19. Jurnal Penelitian Pendidikan.

[CR95] Redecker, C. (2017). *European Framework for the Digital Competence of Educators: DigCompEdu*. JRC Working Papers; Joint Research Centre (Seville site).

[CR96] Robinia KA, Anderson ML (2010). Online Teaching Efficacy of Nurse Faculty. Journal of Professional Nursing.

[CR97] Şad SN, Göktaş Ö (2014). Preservice teachers’ perceptions about using mobile phones and laptops in education as mobile learning tools. British Journal of Educational Technology.

[CR98] Saleh Mahdi H, Sa’ad Al-Dera A (2013). The Impact of Teachers’ Age, Gender and Experience on the Use of Information and Communication Technology in EFL Teaching. English Language Teaching.

[CR99] Salmieri L (2019). The Rhetoric of Digitalization in Italian Educational Policies: Situating Reception among Digitally Skilled Teachers. *Italian Journal of*. Sociology of Education.

[CR100] Sangeeta, & Tandon, U. (2020). Factors influencing adoption of online teaching by school teachers: A study during COVID-19 pandemic. *Journal of Public Affairs.*10.1002/pa.2503 10.1002/pa.2503 PMC7646055 33173442

[CR101] Sari T, Nayır F (2020). Challenges in Distance Education During the (Covid-19) Pandemic Period. *Qualitative*. Research in Education.

[CR102] Scarpellini F, Segre G, Cartabia M (2021). Distance learning in Italian primary and middle school children during the COVID-19 pandemic: a national survey. BMC Public Health.

[CR103] Scherer R, Siddiq F, Tondeur J (2019). The technology acceptance model (TAM): A meta-analytic structural equation modeling approach to explaining teachers’ adoption of digital technology in education. Computers & Education.

[CR104] Schmidt DA, Baran E, Thompson AD, Mishra P, Koehler MJ, Shin TS (2009). Technological pedagogical content knowledge (TPACK) the development and validation of an assessment instrument for preservice teachers. Journal of research on Technology in Education.

[CR105] Selwyn N (1999). Differences in educational computer use: The influence of subject cultures. The Curriculum Journal.

[CR106] Sintema, E. J. (2020). Effect of COVID-19 on the Performance of Grade 12 Students: Implications for STEM Education. *Eurasia Journal of Mathematics, Science and Technology Education, 16*(7). 10.29333/ejmste/7893

[CR107] Stephanou G, Oikonomou A (2018). Teacher Emotions in Primary and Secondary Education: Effects of Self-Efficacy and Collective-Efficacy, and Problem-Solving Appraisal as a Moderating Mechanism. Psychology.

[CR108] Süğümlü, Ü. (2021). A Case Study on Teaching Turkish through Distance Education. *International Journal of Psychology and Educational Studies*, *8*(1), 174–190. 10.17220/ijpes.2021.8.1.278

[CR109] Tajeddin Z., & Alemi, M. (2019). Effective Language Teachers as Persons: Exploring Pre-service and Inservice Teachers’ Beliefs. The Electronic Journal for English as a Second Language 22 (4).

[CR110] Takunyaci, M. (2021). Investigation of Mathematics Teachers’ Self-Efficacy in Teaching Mathematics in the COVID-19 Pandemic Process. *Education Quarterly Reviews, 4*(2). 10.31014/aior.1993.04.02.289

[CR111] Tarchi C, Brante EW, Jokar M, Manzari E (2022). Pre-service teachers’ conceptions of online learning in emergency distance education: How is it defined and what self-regulated learning skills are associated with it?. Teaching and Teacher Education.

[CR112] Tapscott D (2008). *Grown up digital*.

[CR113] Teo T (2009). Modelling technology acceptance in education: A study of pre-service teachers. Computers & Education.

[CR114] Teo T (2011). Factors influencing teachers’ intention to use technology: Model development and test. Computers & Education.

[CR115] Teo, T., & Milutinovic, V. (2015). Modelling the intention to use technology for teaching mathematics among pre-service teachers in Serbia. *Australasian Journal of Educational Technology, 31*(4). 10.14742/ajet.1668

[CR116] Tokmak HS, Incikabi L, Ozgelen S (2013). An Investigation of Change in Mathematics, Science, and Literacy Education Pre-service Teachers’ TPACK. Asia-Pacific Edu Res.

[CR117] Tondeur J, Aesaert K, Prestridge S, Consuegra E (2018). A multilevel analysis of what matters in the training of pre-service teacher’s ICT competencies. Computers & Education.

[CR118] Tondeur J, Petko D, Christensen R, Drossel K, Starkey L, Knezek G, Schmidt-Crawford DA (2021). Quality criteria for conceptual technology integration models in education: Bridging research and practice. Educational Technology Research and Development.

[CR119] Trubavina I, Dotsenko S, Naboka O, Chaikovskyi M, Meshko H (2021). Developing digital competence of teachers of Humanitarian disciplines in the conditions of COVID-19 quarantine measures. Journal of Physics: Conference Series.

[CR120] Tschannen-Moran M, Hoy AW (2001). Teacher efficacy: Capturing an elusive construct. Teaching and Teacher Education.

[CR121] Turner KL, Hughes M, Presland K (2020). Learning Loss, a Potential Challenge for Transition to Undergraduate Study Following COVID19 School Disruption. Journal of Chemical Education.

[CR122] Tzankova, I., Compare, C., Marzana, D., et al. (2022). Emergency online school learning during COVID-19 lockdown: A qualitative study of adolescents’ experiences in Italy. *Curr Psychol*. 10.1007/s12144-021-02674-8 10.1007/s12144-021-02674-8 PMC8739356 35018086

[CR123] Ursavaş ÖF, Yalçın Y, Bakır E (2019). The effect of subjective norms on preservice and in-service teachers’ behavioural intentions to use technology: A multigroup multimodel study. British Journal of Educational Technology.

[CR124] Valtonen T, Kukkonen J, Kontkanen S, Sormunen K, Dillon P, Sointu E (2015). The impact of authentic learning experiences with ICT on pre-service teachers’ intentions to use ICT for teaching and learning. Computers & Education.

[CR125] Voogt J, Erstad O, Dede C, Mishra P (2013). Challenges to learning and schooling in the digital networked world of the 21st century: Learning and schooling in a digital world. Journal of Computer Assisted Learning.

[CR126] Washbon JL (2012). Learning and the new workplace: Impacts of technology change on postsecondary career and technical education. New Directions for Community Colleges.

[CR127] Wong SL, Hanafi A (2007). Gender Differences in Attitudes towards Information Technology among Malaysian Student Teachers: A Case Study at Universiti Putra Malaysia. Educational Technology & Society.

[CR128] Yang J, Wang Q, Wang J, Huang M, Ma Y (2021). A study of K-12 teachers’ TPACK on the technology acceptance of E-schoolbag. Interactive Learning Environments.

[CR129] Yang X (2020). Teachers’ Perceptions of Large-Scale Online Teaching as an Epidemic Prevention and Control Strategy in China. ECNU Review of Education.

[CR130] Zhao Y, Frank KA (2003). Factors Affecting Technology Uses in Schools: An Ecological Perspective. American Educational Research Journal.

